# Development of a Novel Apigenin Dosage form as a Substitute for the Modern Triple Antithrombotic Regimen

**DOI:** 10.3390/molecules28052311

**Published:** 2023-03-02

**Authors:** Antonios D. Tsiailanis, Constantinos C. Tellis, Paraskevi Papakyriakopoulou, Androniki D. Kostagianni, Vasileios Gkalpinos, Christos M. Chatzigiannis, Nikolaos Kostomitsopoulos, Georgia Valsami, Alexandros D. Tselepis, Andreas G. Tzakos

**Affiliations:** 1 Section of Organic Chemistry and Biochemistry, Department of Chemistry, University of Ioannina, 45110 Ioannina, Greece; 2 Atherothrombosis Research Centre, Laboratory of Biochemistry, Department of Chemistry, University of Ioannina, 45110 Ioannina, Greece; 3 Department of Pharmacy, School of Health Sciences, National and Kapodistrian University of Athens, 15771 Athens, Greece; 4 Center for Clinical, Experimental Surgery and Translational Research, Biomedical Research Foundation of the Academy of Athens, 11527 Athens, Greece

**Keywords:** triple antiplatelet activity, flavonoids, DHA, liquid chromatography, pharmacokinetics

## Abstract

The simultaneous administration of three antiplatelet agents has been proposed as an efficient strategy for the secondary prevention of atherothrombotic events and is included in the European guidelines. However, this strategy presented an increased risk of bleeding; therefore, the identification of new antiplatelet agents, with improved efficacy and diminished side effects, is of great importance. In silico studies, UPLC/MS Q-TOF plasma stability, in vitro platelet aggregation experiments, and pharmacokinetic studies were exploited. In the present study, it has been predicted that the flavonoid apigenin could target different platelet activation pathways, including P2Y12, protease-activated receptor-1 (PAR-1), and cyclooxygenase 1 (COX-1). To enhance apigenin’s potency, hybridization with docosahexaenoic acid (DHA) was performed, as fatty acids have illustrated potent efficacy against cardiovascular diseases (CVDs). The new molecular hybrid, termed 4′-DHA-apigenin, demonstrated enhanced inhibitory activity against platelet aggregation induced by thrombin receptor activator peptide-6 (TRAP-6), adenosine diphosphate (ADP), and arachidonic acid (AA), with respect to the parent apigenin. The 4′-DHA-apigenin hybrid illustrated an almost 2-fold enhanced inhibitory activity, with respect to apigenin, and an almost 3-fold enhanced inhibitory activity, with respect to DHA, for the ADP-induced platelet aggregation. Additionally, the hybrid presented a more than 12-fold enhanced inhibitory activity with respect to DHA for the TRAP-6 induced platelet aggregation. Furthermore, a 2-fold enhanced inhibitory activity was recorded for the 4′-DHA-apigenin hybrid for the AA-induced platelet aggregation with respect to apigenin. To surmount the reduced LC-MS based plasma stability, a novel dosage form in olive oil has been developed. The 4′-DHA-apigenin olive oil-based formulation presented an enhanced antiplatelet inhibitory effect in three activation pathways. To further explore the pharmacokinetic profile of 4′-DHA-apigenin in olive oil formulations, a UPLC/MS Q-TOF protocol has been established to quantify the serum levels of apigenin after oral administration to C57BL/6J wild type mice. The olive oil-based formulation of 4′-DHA-apigenin demonstrated an increase in apigenin bioavailability of 262 %. This study may offer a new therapeutic strategy tailored to improve the treatment of CVDs.

## 1. Introduction

Acute myocardial infarction is the most common cause of mortality worldwide [1], accounting 17.9 million deaths each year [2], and the initial cause is the decrease in oxygen supply to the myocardium. This reduction is mostly due to blockage of one or more coronary arteries from ruptures of atherosclerotic plaque. Platelet activation and aggregation play a central role in the development of atherosclerotic plaque and cardiovascular thrombosis [3]. Numerous signaling pathways are involved in the activation of platelets and the subsequent formation of thrombosis. Importantly, platelets have the ability to affect the microenvironment of the plaque, as well as its core stability [4]. As a result, antiplatelet therapy has been utilized to prevent and treat acute myocardial infarction [5]. Currently, a combination treatment of aspirin and clopidogrel (dual antiplatelet therapy) has been shown to be beneficial in patients with acute coronary syndrome (STEMI or NSTEMI) and or stroke [6,7]. Despite the dual antiplatelet therapy, a proportion of patients continues to suffer from thrombotic ischemic events, likely caused by their resistance to aspirin or/and clopidogrel. In addition, it is known that dual antiplatelet therapy is related to bleeding complications, leading to limitations in their application. This is due to the fact that each agent can selectively inhibit one receptor in the platelet, resulting in limited application efficacy [8].

Recently, a triple antiplatelet therapy for the secondary prevention of atherothrombotic events has been established by simultaneously using three antiplatelet agents: a TxA2 inhibitor (aspirin), a P2Y12 receptor antagonist (clopidogrel), and a PAR-1 antagonist (vorapaxar) [9]. Nevertheless, a significant number of patients continue to experience cardiovascular events, and it is reported that triple antithrombotic therapy increases the bleeding risk compared with anticoagulation or dual antiplatelet therapy alone [10,11,12,13]. Therefore, there is an urgent need to enrich the established antiplatelet therapies with new bioactive molecules. Those molecules should be designed to have antithrombotic efficacy, with no combination bleeding effects, so as to provide a new therapeutic approach for a well-balanced therapy between thrombosis and bleeding. This could be achieved by discovering new therapeutic agents that possess multiple inhibitory effects against platelet activation. Our research group recently evaluated the antiplatelet activity profile of hexane olive leaf extract in human platelets and demonstrated a strong antiplatelet activity that inhibited platelet activation induced through both P2Y12 and PAR1 receptors [14]. In this context, our group developed a novel molecular hybrid that inhibited platelet aggregation by conjugating the flavonoid naringenin to the omega-3 fatty acid DHA [15].

Natural products have served as a rich source of bioactive molecules. Many drugs that have been developed from medicinal plants have illustrated satisfactory efficacy and low toxicity, or have indirectly served as scaffolds for the synthesis of novel, more potent substances [16]. More specifically, flavonoids present a wide range of beneficial results, including anti-inflammatory, anti-angiogenic, and antiplatelet effects. Epidemiological studies have shown that the consumption of a flavonoid-rich diet significantly reduces the risk of atherosclerotic cardiovascular disease (ASCVD) [17,18]. Flavonoids present antiaggregatory activity and are currently considered to be an important area in the drug discovery of anti-thrombotic agents. Numerous studies have been carried out to identify the key structural features that give flavonoids their antiplatelet properties [19,20]. Apigenin is a flavone that possesses the ability to inhibit platelet adhesion [21]. Specifically, apigenin was found to modulate signaling through binding to the TxA2 receptor [22,23]. Apigenin has also been suggested to inhibit both PAR1- and PAR4-mediated platelet aggregation, whereas it is a weaker inhibitor of thrombin-induced aggregation [20]. Nonetheless, apigenin is characterized by reduced bioavailability, mainly due to its polar phenol groups, which could reduce its transporting efficacy through the lipophilic cell membrane thus reducing its antiplatelet potency [24]. Along these lines, different prodrugs have been developed to enhance the activity of flavonoids, as well as their stability and their cell uptake [15,25,26,27].

Epidemiological studies have shown that the consumption or supplementation of polyunsaturated fatty acid (PUFAs) significantly reduces the risk of ASCVD [28,29,30]. DHA consists of the primary omega-3 fatty acids in fish oil. DHA exerts cardioprotective effects, improving cardiovascular function in terms of anti-inflammatory effects on peripheral artery disease. It reduces major cardiovascular events via antiplatelet and anticoagulant effects, and it is used as a subtherapeutic to anticoagulation therapies in case of aspirin resistance or clopidogrel hypo responsiveness [31,32,33]. Although PUFAs are not able to directly change the platelet aggregation, they seem to facilitate aspirin-induced inhibition. However, it has been suggested that the presence of, or changes in, the composition ω-3 PUFA—which is incorporated into platelet membranes—can alter not only the permeability, but also the function and activity of receptors and transporters inducing platelet responses [32], the mechanisms of the antithrombotic effects of omega-3 PUFAs still remain unclear.

Therefore, in the present study, is hypothesized that conjugating apigenin with DHA could lead to a new single entity with an enhanced pharmacological profile. This could be attributed to the fact that DHA could increase the hydrophobicity of apigenin, resulting in enhanced cell permeability. In addition, the tethering of DHA with apigenin could lead to the inhibition in all three platelet aggregation pathways triggered by ADP, AA, and TRAP-6 [15].

Towards this end, a new molecular hybrid that could present enhanced antiplatelet activity with respect to the parent compound apigenin was rationally developed. Afterward, the synthesis of the hybrid was followed up with the evaluation of its inhibitory activity against platelet aggregation induced by three different platelet agonists (ADP, TRAP-6, and AA). The establishment of UHPLC-MS/MS assays was necessary in order to evaluate the in vitro stability of the hybrid in human plasma. To enhance the bioavailability of the hybrid, in the current study, a new dosage form was developed using olive oil as a vehicle. The new formulation demonstrated enhanced antiplatelet activity. Finally, serum pharmacokinetics of both the apigenin hybrid and the apigenin parent (native) compound, formulated as olive oil-based dosage forms, were evaluated in a C57BL/6J mice model.

## 2. Results and Discussion

### 2.1. Rational Design and 4′-DHA-Apigenin

**Figure 1:**
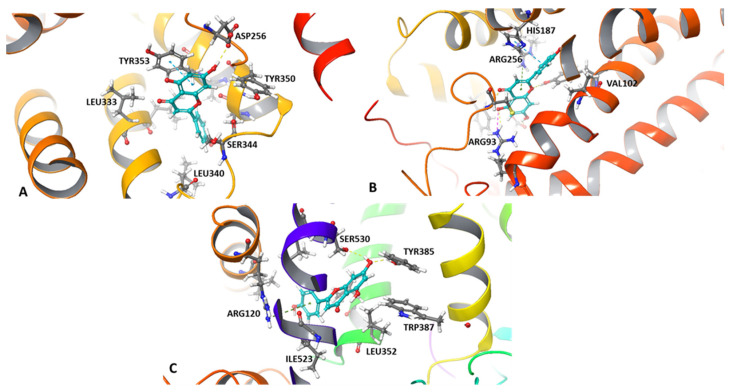
Most favorable docked poses of apigenin in the three receptors and their hydrogen and pi interactions (**A**) PAR-1, (**B**) P2Y12, and (**C**) COX-1.

Natural products can be exploited to combat an array of diseases, including cancer, inflammation, and atherosclerosis, due to their antioxidant properties [2]. Flavonoids have been identified to adopt antiplatelet activity at submicromolar concentrations [19]. This has suggested that a dietary consumption of flavonoids could have a therapeutic potential and could impact the in vivo aggregation of platelets. In another study, the antiplatelet effect of several natural flavonoids was evaluated [34]. It was identified that the isoflavonoids genistein and daidzein adopted a marked COX-1 inhibitory activity. Several synthetic approaches have been followed to enhance the antiplatelet activity of flavonoids [35]. It was found that neither the carbonyl group, nor the ring B phenyl groups, are particularly important for the expression of the antiplatelet activity of these flavonoids. In another study, it was found that the synthetic analogues of regioselectively acylated quercetin, quercetin-3-O-propionate, and quercetin-3-O-butyrate were more effective in inhibiting platelet aggregation with respect to quercetin [36]. These studies indicate that the flavonoid structures can be exploited towards the development of novel potent scaffolds to inhibit platelet aggregation. Apigenin, as one of the most abundant natural products, was selected based on its ability to inhibit platelet aggregation [17]. To further confirm the antiplatelet properties of apigenin, docking calculations were conducted to explore the interactions of apigenin with each receptor. Apigenin was placed in the same location in PAR-1 as vorapaxar, a known selective inhibitor and anti-platelet drug. As it shown in Figure 1, apigenin develops hydrogen bonds with ASP 256, pi interactions with TYR 353, and hydrophobic interactions with several residues (Table S1). The Glide XP score is −8.57 kcal/mol. For the P2Y12 and the COX-1 receptors, apigenin binds in the same location as ADP and celecoxib—a nonsteroidal anti-inflammatory drug (NSAID)—respectively (Figure 1B,C). Apigenin forms multiple hydrogen bonds with the P2Y12 residues CYS 175, GLU 281, ARG 93, TYR 105, and pi interactions with HIS 187 and ARG 256. The Glide XP score is −4.75 kcal/mol, and the potential energy (OPLS 3) is −586.52 kcal/mol. Concerning receptor COX-1, the natural product interacts via hydrogen bonds with TYR 385, SER 530, via pi interactions with ARG 120 and forms several hydrophobic interactions with multiple residues (Table S1).

The above in silico results indicate the potential of apigenin to bind efficiently to the three major receptors implicated in platelet aggregation. Consequently, apigenin can operate as a candidate so as to develop a novel hybrid that can simultaneously target PAR-1, P2Y12, and COX-1, achieving triple antiplatelet therapy.

Our scientific group then proceeded to determine the site that can be utilized for bioconjugation with the DHA. Apigenin demonstrated not only poor penetration through the lipophilic cell membrane, but also low stability, due to the presence of hydroxyl groups [32]. Specifically, their phenolic functional groups were implicated in extensive metabolism, where transformations such as glucuronidation, sulfonation, or methylation take place [33]. In addition, it has been reported that ring B is less critical for the antiplatelet activity of flavonoids; thus, it could be a possible location for modifications [35].

**Figure 2:**
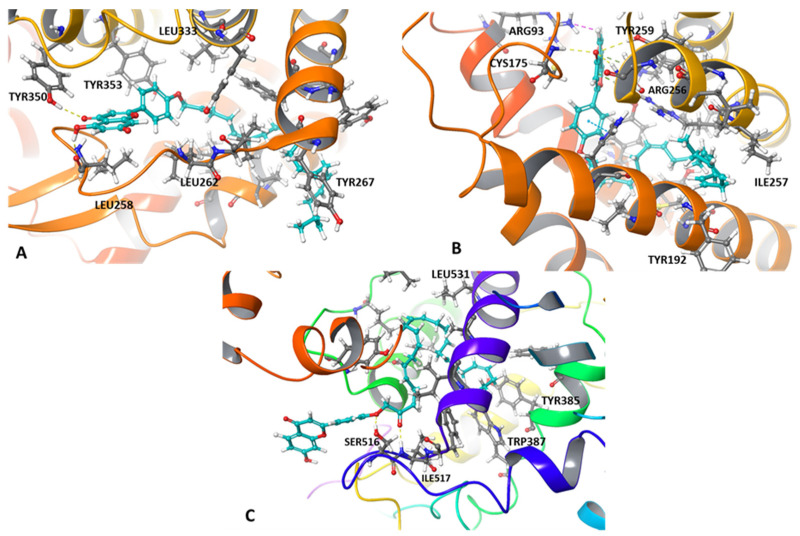
Most favorable docked poses of 4′-DHA-apigenin in the three receptors and their hydrogen and pi interactions (**A**) PAR-1, (**B**) P2Y12, and (**C**) COX-1.

Thus, the 4′ OH phenolic group of ring B was selected to conjugate the DHA. After having determined the most preferential conjugation site, we employed in silico studies to investigate whether the designed hybrid would retain the binding affinity in all platelet activation pathways. Therefore, in silico studies were performed between 4′-DHA-apigenin and the three platelet activation pathways: COX-1, PAR-1, and P2Y12. The 4′-DHA-apigenin performs multiple favorable interactions with each receptor, forming more stable docking poses, with lower potential energy, than the parent compound apigenin. Specifically, 4′-DHA-apigenin forms hydrogen bonds with PAR-1 residues TYR 350, LEU258, and TYR 337 (Figure 2A and Table S2). The new modified molecule is stabilized in the receptor with two more hydrophobic interactions, and the docking score is improved (−13.461 kcal/mol) compared to apigenin (−8.57 kcal/mol).

Concerning receptor P2Y12, the new compound forms hydrogen bonds with ARG 93, CYS 175, GLN 263, and TYR 259, and pi interactions with LYS 280, ARG 256, and HIS 187 (Figure 2B). The 4′-DHA-apigenin is stabilized with five more hydrophobic interactions in the receptor, lowering the potential energy at −828.489 kcal/mol compared to −586.52 kcal/mol that apigenin exhibited for P2Y12. Figure 2C illustrates the docking pose of 4′-DHA-apigenin forming hydrogen bonds with residues ILE 517, and SER 516 and forms five more hydrophobic interactions with COX-1. The potential energy improved significantly compared to that of the parent compound apigenin (Table S2).

From the docking results, it is evident that the new hybrid of apigenin could illustrate an improved profile in its antiplatelet activity, and it may be used as a triple antiplatelet agent.

### 2.2. Chemical Synthesis of the Molecular Hybrid

**Scheme 1:**
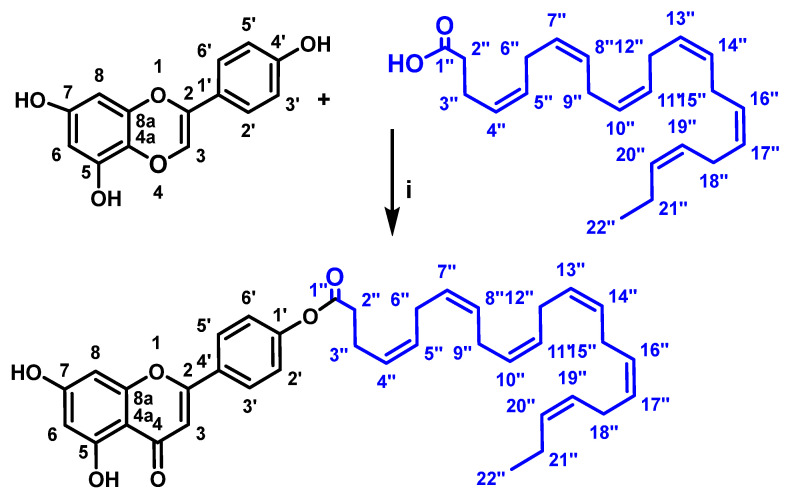
Chemical synthesis of the 4′-DHA-apigenin hybrid. Reagents and conditions: (i) DCC, DMAP, THF, rt, 2 h.

**Figure 3:**
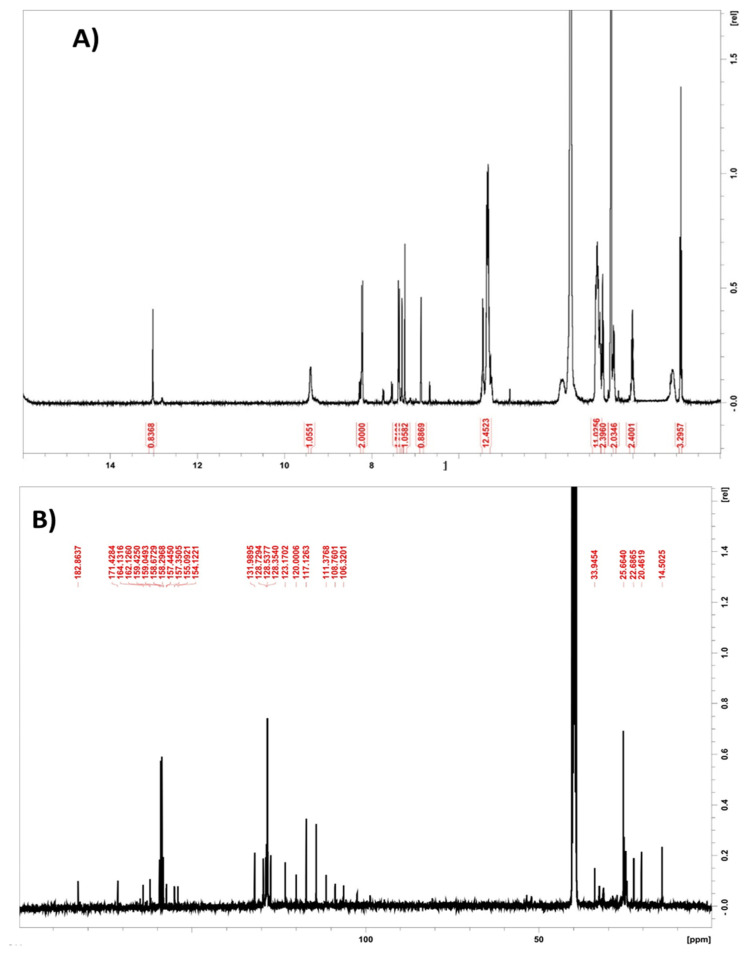

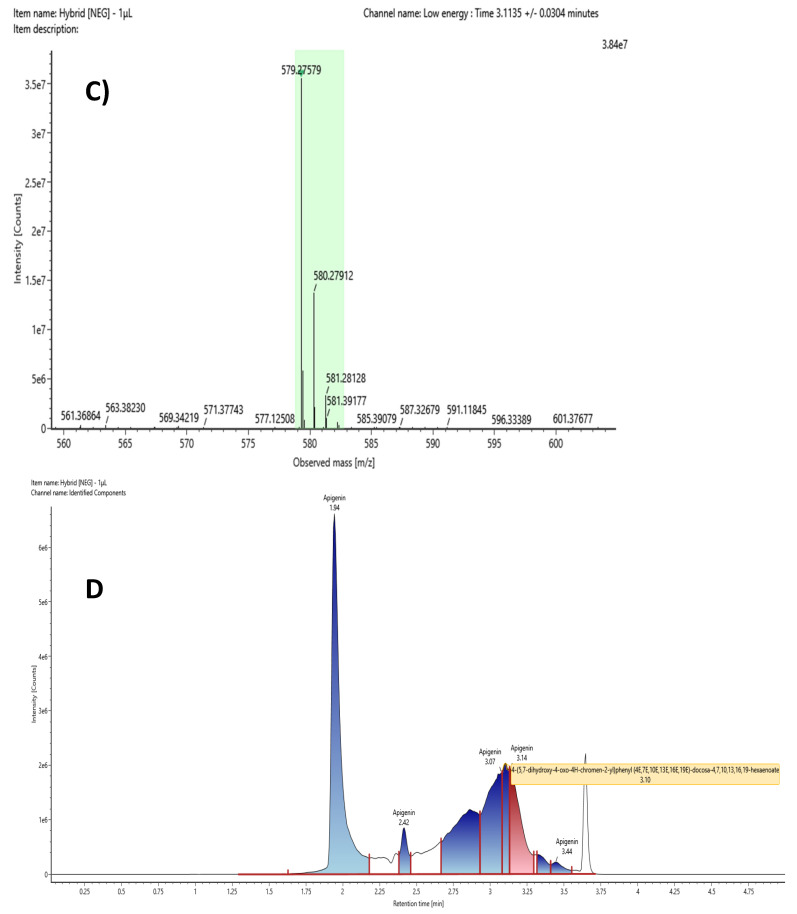
(**A**,**B**) ^1^H and ^13^C NMR spectrum, respectively, of 4′−DHA−apigenin in DMSO−d_6_ (θ = 25 °C) (**C**,**D**) HRMS spectrum and chromatogram of 4′−DHA-apigenin; *m*/*z* 579.27.

Driven by the in silico studies, the synthesis of the 4′-DHA-apigenin hybrid was performed as shown in Scheme 1. The designed hybrid was achieved through the coupling of the carboxylic acid of DHA and the phenolic group of apigenin using DCC and DMAP (Steglich esterification), under inert conditions. In the 13C NMR of the compound peaks 182.8637 ppm and 171.4284 ppm are evidence of carbon 1″ and 4″, respectively; peaks resulting from carbons 2″, 3″, 6″, 9″, 12″, 15″, 18″, 21″, and 22″ can be seen between 10 and 40 ppm, as they are more protected, while peaks from carbons 4′, 3′, 5′, 4″, 5″, 7″, 8″, 10″, 11″, 13″, 14″, 16″, 17″, 19″, and 20″ possibly overlap at the 123–132 ppm fields. Peaks at 106.3201 ppm and 108.7601 ppm possibly correspond to carbons 8 and 6, respectively (Figures S1 and S2) The LC-MS data can be seen in Figure 3C (the centroid masses) and Figure 3D the chromatogram of 4′-DHA-apigenin.

### 2.3. Evaluation of the Antiplatelet Activity of Different Agents against ADP, AA, and TRAP-6

The activity of the hybrid was evaluated in vitro in platelet aggregation induced through P2Y12 and PAR-1 activation (using ADP and TRAP-6 as agonists, respectively), as well as through the COX-1 pathway, using AA as an agonist. The 4′-DHA-apigenin hybrid significantly inhibited platelet aggregation induced by all agonists in a dose-dependent manner, exhibiting IC50 values of 353 ± 36 μΜ for AA, 362 ± 20 μΜ for ADP, and 52 ± 12 μΜ for TRAP-6 (Table 1).

**Figure 4:**
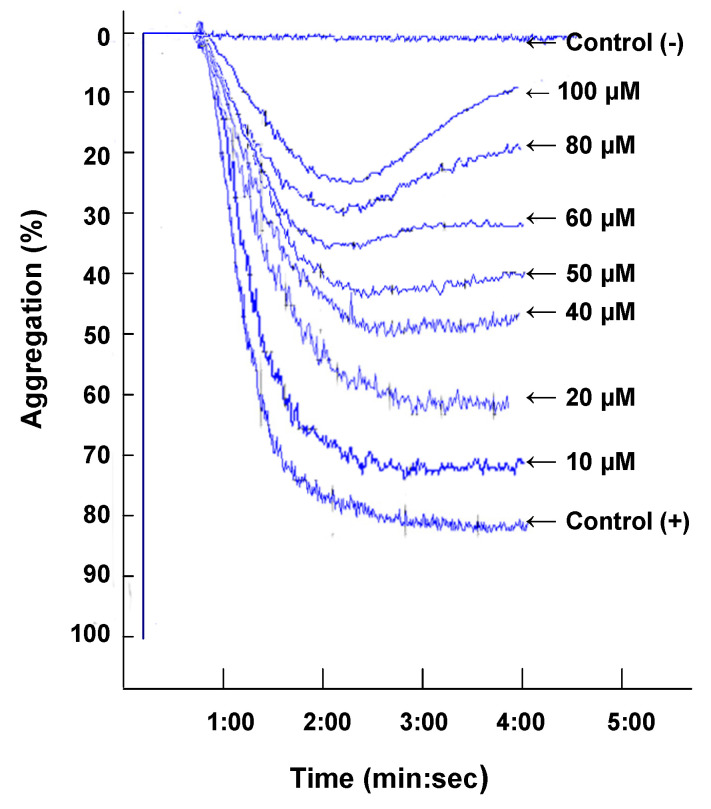
Representative aggregation curves illustrating the inhibitory effect of different concentrations of 4′−DHA-apigenin hybrid on platelet aggregation induced by TRAP−6.

As it is shown in Table 1, the 4′-DHA-apigenin hybrid exhibited an increased inhibitory effect on platelet aggregation induced by all agonists when compared to its conjugates. The IC50 value of the 4′-DHA-apigenin hybrid was significantly lower compared with the equimolar solution of apigenin/DHA when TRAP-6 and ADP were used as a platelet agonists (52 ± 12 v/s 610 ± 40, *p* < 0.005) and (362 ± 20 v/s 580 ± 39, *p* < 0.005), respectively, but it was higher towards AA-induced platelet aggregation (353 ± 36 v/s 210 ± 25, *p* < 0.005). The IC50 value of the 4′-DHA-apigenin hybrid was significantly lower when compared with apigenin on platelet aggregation induced by ADP (362 ± 20 v/s 607 ± 35, *p* < 0.005) or AA (353± 36 v/s 850 ± 45, *p* < 0.005), while no significant differences were observed between the 4′-DHA-apigenin hybrid and apigenin towards TRAP-6-induced platelet aggregation (52 ± 12 v/s 56 ± 8). Figure 4 illustrates representative aggregation curves of the dose-dependent inhibitory effect of the 4′-DHA-apigenin hybrid on TRAP-6-induced platelet aggregation. The IC50 value of the 4′-DHA-apigenin hybrid was significantly lower when compared with DHA on platelet aggregation -induced by ADP (362 ± 20 v/s 920 ± 53, *p* < 0.005) and TRAP-6 (52 ± 12 v/s 680± 42, *p* < 0.005), respectively, while it was higher when compared with DHA (362 ± 20 v/s 130 ± 18). on platelet aggregation -induced by AA None of the compounds tested, at a concentration of 500 μM, exhibit platelet aggregatory activity during 5 min incubation with PRP.

### 2.4. Evaluation of Stability of the 4′-DHA-Apigenin Hybrid in Human Plasma

**Figure 5:**
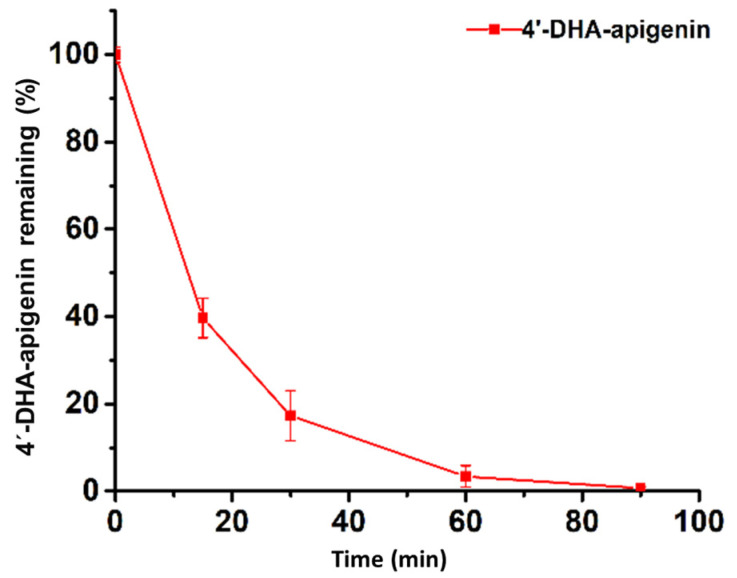
Degradation rate of 4′-DHA-apigenin hybrid during incubation in human plasma for 90 min.

The in vitro stability of 4′-DHA-apigenin hybrid in human plasma was evaluated. To achieve this, the establishment of LC-MS/MS protocols to monitor its stability was required. During chromatographic optimization, several parameters were evaluated, including different mobile phases, column dimensions, and various ratios of the mobile phases to achieve optimum peak shape and short running times. The negative electrospray ionization mode was selected in MS, based on the structure of the hybrid. Utilizing MRM builder, a feature of MSWS software, the most abundant transitions in terms of sensitivity were *m*/*z* 579.3 → 267.9 and 579.3 → 403. The degradation rate of 4′-DHA-apigenin, after incubation in human plasma at 37 °C for 0, 15, 30, 60, and 90 min, is presented in Figure 5. The elimination rate was rather fast, since only 17.3% of the parent compound was still present after 30 min of incubation, with a t_1/2_ of approximately 13 min.

### 2.5. Preparation of 4′-DHA-Apigenin Olive Oil-Based Formulation

To optimize the clinical potential of this hybrid, it is of importance to develop a novel formulation to overcome the problems such as its recorded low plasma stability and its oral bioavailability. In recent years, lipid-based formulations have attracted the interest of the research community, as these formulates can enhance the stability of drugs in human plasma, as well as their oral availability, leading to the successful development of medicinal products. Olive oil can be used as a vehicle for drugs that exhibit poor water solubility and low stability. Moreover, due to the beneficial effects exerted by olive oil, it can contribute to the prevention, development, and progression of CVDs. Thus, our research group proceeded with the development of an olive oil-based formulation of the generated 4′-DHA-apigenin molecular hybrid.

### 2.6. In Vitro Antiplatelet Activity of the 4′-DHA-Apigenin Formulation

**Figure 6:**
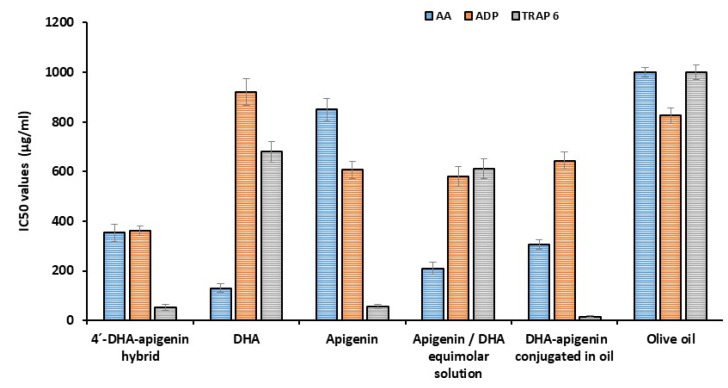
IC50 values of the inhibitory effect of 4′-DHA-apigenin hybrid, DHA, apigenin, apigenin/DHA equimolar solution, 4′-DHA-apigenin hybrid olive oil formulation, and olive oil on platelet aggregation induced by ADP, AA, or TRAP-6 in PRP. Bars represent the mean ± SD.

In the current study, the exploitation of the antiplatelet activity of the 4′-DHA-apigenin formulation was needed. Interestingly, we recorded that the developed formulation was able to significantly inhibit platelet aggregation induced by the three utilized platelet aggregation agonists with respect to the 4′-DHA-apigenin hybrid. Remarkably a higher than 3-fold reduction was recorded on the IC50 induced platelet aggregation by TRAP-6 (16 ± 3 v/s 52 ± 12) for the 4′-DHA-apigenin hybrid olive oil formulation with respect to the unformulated 4′-DHA-apigenin hybrid (Table 2 and Figure 6).

### 2.7. Pharmacokinetic Studies of Native Apigenin and 4′-DHA-Apigenin Olive OilBased Formulation in C57BL/6J Mice

**Figure 7:**
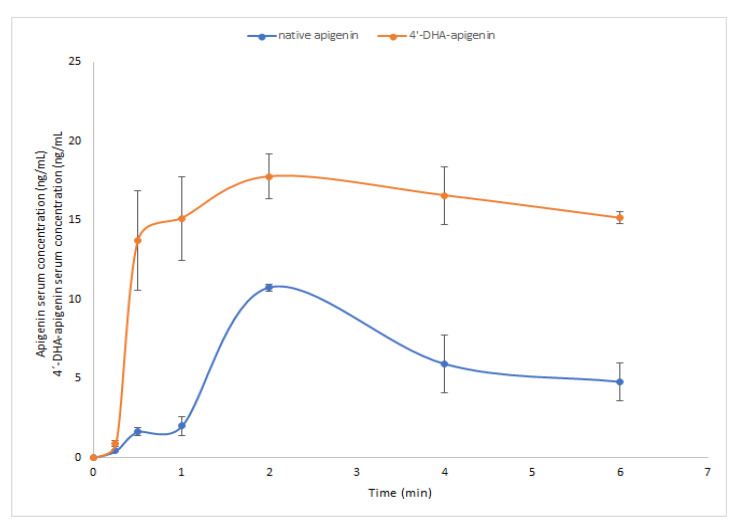
Pharmacokinetic profile of native apigenin and 4′-DHA-apigenin in serum, after oral administration in mice, using olive oil as the vehicle (error bars represent ± SD of measured apigenin and 4′-DHA-apigenin concentration).

Having determined that the new formulation of 4′-DHA-apigenin delivered a beneficial pharmacological profile, comparative pharmacokinetic (PK) study of the native apigenin and the 4′-DHA-apigenin olive oil formulation was then performed in C57BL/6J mice after oral administration. A UPLC/MS Q-TOF bioassay method was established to determine apigenin and 4′-DHA-apigenin in C57BL/6J mice serum. The serum concentration of apigenin and 4′-DHA-apigenin, at all sampling times after oral administration, were quantified and are presented in Figure 7. Mice were administrated with apigenin (dissolved in extra virgin olive oil) and an equimolar dose of the 4′-DHA-apigenin olive oil-based formulation, and blood was collected at 15, 30, 60, 120, 240, and 360 min after administration. Based on the PK profiles in Figure 7, 4′-DHA-apigenin appears to be absorbed more rapidly and more excessively than native apigenin, while the serum concentration remains almost constant at C_max_ for the 6 h period of the study. Native apigenin reached C_max_ at 2 h after oral administration, and it declined progressively during the following 4 h of the study. Based on the calculated % of relative bioavailability, 4′-DHA-apigenine was found to be significantly more bioavailable —specifically, 262% more—than native apigenin after oral administration. The estimated pharmacokinetic parameters of native apigenin and 4′-DHA-apigenin are summarized in Table 3. It should be mentioned that the calculated AUC_inf_ values should be considered with caution, since the percentage of extrapolation based on the calculated λ_z_ value was 40.5% and 80.5% for native apigenin and 4′-DHA-apigenin, respectively.

However, the liquid nature of the prepared 4′-DHA-apigenin olive oil-based formulation may limit its use. Accordingly, the optimization of the composition of the prepared olive oil-based formulation is challenging. For example, the incorporation of an emulsifier, such as the biocompatible polymer hydroxypropyl-methyl cellulose (HPMC), would enable the use of freeze-drying techniques to prepare solid formulations, with enhanced storage stability and bioavailability characteristics.

## 3. Materials and Methods

### 3.1. Chemicals and Reagents

Cis-4,7,10,13,16,19-Docosahexanoic acid (DHA) and apigenin were purchased from Carbosynth Ltd. Formic acid (98%, LC-MS grade) was obtained from Fluka. Methanol, water, LC-MS grade, as well as N, N’-dicyclohexylcarbodiimide (DCC), dimethylaminopyridine (DMAP), methanol, dichloromethane, acetonitrile, HPLC grade all, and trifluoroacetic acid (peptide synthesis grade) were obtained from Fisher Scientific. TLC of the highest available purity was carried out on silica gel plates (UV254) obtained from Alfa Aesar. DMSO (LC-MS grade) was purchased from Thermo Scientific. DMSO-d_6_ (99.8%) was purchased from Euriso-top. NMR experiments were performed on a Bruker Avance 400 MHz spectrometer equipped with a z-gradient unit (Bruker BioSpin, Rheinstetten, Germany). Membrane filters with a 0.2 μm pore size and a 4 mm diameter were purchased from Phenomenex. High purity argon was used as a collision gas. ADP was obtained from Chrono-Log Corp (Havertown, PA, USA). AA was purchased from Sigma-Aldrich, St. Louis, MO, USA, and TRAP-6 was obtained from Bachem, Bubendorf, Switzerland. For the stability studies, drug-free human plasma from healthy donors was kindly offered from the Blood Donation Center of the University Hospital of Ioannina.

### 3.2. Synthesis of the 4′-DHA-Apigenin Hybrid

To synthesize 4′-DHA-apigenin, apigenin (9.54 mg, 0.035054 mmol) was added to the solution of DHA (11.6 μL, 0.0338535 mmol) in 2 mL anhydrous tetrahydrofuran under inert atmosphere. After 5 min, a solution of N, N’-Dicyclohexylcarbodiimide DCC (9.8 mg, 0.04408 mmol) and a catalytic amount of 4-Dimethylaminopyridine (DMAP) in 2 mL anhydrous tetrahydrofuran was added dropwise, and the reaction was continued at room temperature for 2 h. TLC illustrated the formation of a new spot. The solvent was evaporated on a rotary evaporator, and the residue was purified by RP-HPLC using H_2_O × 0.1%TFA/MeCN × 0.1%TFA as eluents to yield 13.1 mg (65.9%) of a light yellow solid. ^1^H-NMR of 4′-DHA-apigenin: ^1^H-NMR (400 MHz, DMSO-d_6_, 25 °C): δ = 13.03 (s, 1 H, 5-OH), 9.39 (s, 1H, 7-OH), 8.24–8.20 (d, 2H, 2′-H, 6′-H), 7.38–7.35 (d, 2H, 3′-H, 5′-H), 7.30 (d, 1H, H-8), 7.23 (s, 1H, 3-H),6.86 (d, 1H, H-6), 5.35–5.30 (m, 12 H, D4), 2.82–2.79 (m, 11H, D5,3-Ha), 2.69 (t, 2H, D6), 2.43 (q, 2H, D8), 2.01 (m, 2H, D2), 0.89 (t, 3H, D1) ppm;^13^C-NMR of 4′-DHA-apigenin: (100 MHz, DMSO-d_6_, 25 °C): δ = 182.1, 171.4, 164.13, 162.13, 159.42, 159.04, 158.67, 158.29, 157.44, 131.98, 129.50, 128.72, 128.35, 128,32, 123.17, 117.12, 111.37, 108.76, 106,32, 33.94, 25.66, 22.68, 20.46, 14.5 ppm. Mass: MS (ESI) *m*/*z*: [M-H]^-^ for C_37_H_40_O_6_: calc: 579.28, found: 579.27.

### 3.3. Platelet Aggregation Studies of Apigenin and Its Conjugates

The antiplatelet activity of DHA-apigenin, apigenin, DHA, and equimolar solution of apigenin/DHA was studied in platelet rich plasma (PRP) by light transmittance aggregometry (LTA) assay, as previously described [14,15,37,38]. Briefly, PRP was prepared from peripheral venous blood of apparently healthy normolipidemic volunteers. The platelets of PRP were adjusted to a final concentration of 2.5 × 10^8^ per Ml with homologous platelet-poor plasma (PPP). The PRP was then pre-incubated with each one of the compounds for 1 min before the initiation of aggregation. The final DMSO concentration in each assay was <0.5% (*v*/*v*). In the control experiments, the PRP was incubated with each one of the compounds for 5 min in order to evaluate whether each tested compound was a platelet activator. Platelet aggregation induced by ADP (10 Μm), AA (500 Μm), and TRAP-6 (10 Μm) was determined in aliquots of 0.5 Ml of PRP, at 37 °C under continuous stirring at 1200 rpm, in a Chronolog Lumi-Aggregometer (model 700 4-channel) equipped with the AggroLink software package. The maximal aggregation, achieved within 4 min after the addition of each agonist, was determined and expressed as a percentage of 100% light transmission calibrated for each specimen (maximal percentage of aggregation; MPA). Τhe inhibitory efficacy of each compound tested was also expressed as IC50 values (concentration that induces 50% inhibition of platelet aggregation). All aggregation studies were conducted within 3 h after blood was drawn. The inhibition of platelet aggregation (IPA) values induced by each compound was calculated using the IPA (%) = MPA × 100/MPA formula.

### 3.4. LC-MS/MS Studies

#### 3.4.1. Liquid Chromatography and Mass Spectrometry Operational Conditions

Liquid chromatography was performed using an Advance Ultra High Performance Liquid Chromatography (UHPLC) system (Bruker, Germany). The column oven was set at 40 °C. For the separation, a Kinetex C18 column 100 mm × 2.1 mm, 2.6 μm, with a 2.1 mm pro-guard column, was used (Phenomenex). The mobile phases were composed of water (LC-MS grade) with formic acid 0.1% (A) and MeOH (B). The gradient followed at a constant flow of 300 mL/min was: initial phase (B) concentration 5%, increased to 100% within 1 min, then kept constant for 5.5 min, and reduced to 5% until the end of the run. The analysis run time was 7 min, and the RT of the 4′-DHA-apigenin conjugate was 3.8 min. The injection volume was 2 μL, while the auto-sampler temperature was 15 °C.

For the detection of the conjugate, the EVOQ Elite ER TQ (Bruker) mass spectrometer was operated in negative ionization electrospray mode (ESI) under multiple reaction monitoring (MRM). Using MRM builder, a feature of MS workstation Bruker software, the optimal MRM transitions for monitoring 4′-DHA-apigenin were *m*/*z* 579.3 → 267.9 and 579.3 → 403. Optimum ESI conditions were determined as follows: spray voltage, 4500 V; heated probe gas flow, 50 units; heated probe temperature 350℃; cone gas flow, 20 units; cone temperature, 200 °C; nebulizer gas flow, 40 units. Total control of LC and MS, as well as data acquisition, were performed with MSWS software, Version 8.2.1 (Bruker). For the pharmacokinetic studies, a Xevo-G2-XS-QtoF mass spectrometer, coupled to a Waters UPLC I-Class Binary Solvent Manager (Waters Corp., Milford, MA, USA) was used. The mobile phases were composed of water (LC-MS grade) with formic acid 0.1% (A), and acetonitrile (LC-MS grade) with formic acid 0.1% (B). A constant flow of 250 mL/min was used, with an injection volume of 1 μL; the gradient was as follows: initial phase (B) concentration 20%, increased to 100% within 1.5 min, then kept constant for 1 min, and reduced to 20% until the end of the run (4 min total runtime). The MS conditions were as follows: the scan range was set at *m*/*z* 100–600, and the source voltage was 0.8 Kv in negative ionization electrospray mode (ESI). The source temperature was 550 °C, the flow of desolvation gas (N2) was set to 1000 L per hour, and the cone gas flow was set to 20 L per hour. For MS/MS, the collision energy ramp was set from 20 eV to 40 eV, and the declustering potential was 40 V. The column used was the Acquity UPLC^®^ BEH C18 1.7 µm device. For the post processing and analysis of the acquisition data, UNIFY soFTware was used.

#### 3.4.2. Human Plasma Stability Assay

The in vitro stability of the apigenin molecular hybrid was evaluated in human plasma (Ph was adjusted to 7.4) at 37 °C. Stock solution of 1 mg/mL of 4′-DHA-apigenin was prepared by dissolving the appropriate amount of hybrid in MeOH. This stock solution was further diluted to a final working concentration of 50 μM. The assay was conducted in a shaking water bath at 37 °C, and all samples were studied in triplicate. In 90 μL of human plasma, 10 μL of 4′-DHA-apigenin (50 μM stock) were added and incubated for 0, 15, 30, 60, and 90 min at 37 °C. In order to stop the reactions, 300 μL of cold acetonitrile were added. The samples were vortex-mixed and centrifuged at 10,000× *g* for 5 min. Then, the supernatant was taken, filtered, and transferred to vials for LC-MS analysis. The plot of the % faction of the parent compound against time was designed, while the in vitro plasma half-life (t_1/2_) of the hybrid was determined by the equation t_1/2_ = 0.693/b, where b represents the slope in the linear fit of the natural logarithm of the fraction of the parent compound remaining against time.

### 3.5. Formulation of the Hybrid

The 4′-DHA-apigenin hybrid was dissolved in olive oil. The solution was sonicated for 5 min, followed by homogenization by vortexing for 15 min. The solution was stirred at rt for 2 h under inert atmosphere in the dark. The contents were filtered through a nylon filter with a 0.45 μm pore size and then allowed to rest for 3 h before any further measurements.

### 3.6. In Vivo Pharmacokinetic Study

#### 3.6.1. Animals

All animal experiments were performed in the animal facility of the Center of Clinical Experimental Surgery and Translational Research of the Biomedical Research Foundation of the Academy of Athens. The facility is registered as a “breeding and experimental” facility, according to the Greek Presidential Decree 56/2013 and the EC Directive 2010/63 on the Protection of Animals used for Experimental and Other Scientific Purposes (European Union, 2010) (Directive 2010/63/EU). Animals were housed in individually ventilated cages (Techniplast, Varese, Italy) under specific pathogen-free and constant environmental conditions (12:12 h light:dark cycle, 22 ± 2 °C, relative humidity 45–10%), fed on irradiated pellets (4RF22, Mucedola, Milano, Italy), and had access to tap water ad libitum. The cages and bedding were changed once a week. All mice in the facility were screened regularly, according to a health-monitoring program, complying with the recommendations of the Federation of European Laboratory Animal Science Associations. The study was approved by the Veterinary Authorities of the Region of Athens, Greece (300641/15-04-21).

#### 3.6.2. Pharmacokinetic Study Protocol

60 male 8-week-old C57BL/6J wild type mice, with mean weight equal to 23.5 ± 2.1 g were randomly divided in two groups (a, b) of 30 and each one received different treatment as follows: (a) the apigenin hybrid formulation and (b) the parent apigenin, dissolved in extra virgin olive oil, orally via the gavage technique at the dose of 50 mg/kg. Mice in each group were randomly further divided in 6 subgroups of five, each group representing one sampling time point (15, 30, 60, 120, 240, and 360 min). Animals were anaesthetized with sevoflurane, and sacrificed to collect blood samples via cardiac puncture, in non-heparinized Eppendorf ^®^ Tubes, at the time point of interest after administration. Blood samples were centrifuged (10,000× *g* rpm, 15 min, 4 °C), and serum was collected, frozen and stored at −70 °C until further processing. In order to quench the reactions, 395 μL of MeOH were added to each sample, along with IS (5 μL of Rosmarinic acid). The samples were vortexed and centrifuged at 10,000× *g* for 10 min. Then, the supernatant was taken, filtered and transferred to vials for analysis, as described above.

#### 3.6.3. Pharmacokinetic Analysis

The *Phoenix*^TM^ (www.certara.com)^®^. software was used for data analysis using non-compartmental PK analysis (NCA). More specifically, sparse sampling NCA was performed to determine basic serum PK parameters, namely AUC_0−t_, AUC_inf_, C_max_ and T_max_, and to calculate the relative oral bioavailability of apigenin after apigenin hybrid formulation and native apigenin oral administration. To calculate the PK parameters and their standard errors (SE), the mean concentration curve is calculated and used in conjunction with the available subject information. The log-linear trapezoidal method was used to calculate AUC_0−t_ and AUC_inf_, with extrapolation to infinity by dividing the last concentration by the terminal slope, λ_z_ (k_el_, estimated by linear regression analysis on the last three points of the log-transformed concentration vs. time plot). The elimination half-life, t_1/2_, was calculated as t_1/2_ = 0.693/λ_z_ and the relative oral bioavailability (F) of 4′-DHA-apigenin (from apigenin hybrid formulation), compared to native apigenin, was estimated using the following equations:

(1) $$F\;=\frac{{\mathrm{AUC}}_{0-t}{}_{\left(\mathrm{hybrid}\right)}\times {\mathrm{Dose}}_{\left(\mathrm{native}\right)}}{{\mathrm{AUC}}_{0-t\left(\mathrm{native}\right)}\times {\mathrm{Dose}}_{\left(\mathrm{hybrid}\right)}}$$

(2) $$F\;=\frac{{\mathrm{AUC}}_{0-t}{}_{\left(\mathrm{hybrid}\right)}}{{\mathrm{AUC}}_{0-t\left(\mathrm{native}\right)}}$$

where AUC_0−t(hybrid)_ and AUC_0−t(native)_ is the area under the concentration–time curve from time 0 to the last sampling time after oral administration of the apigenin hybrid formulation and native apigenin, respectively; Dose_(hybrid)_ and Dose_(native)_ is the administered dose, which was the same for the apigenin hybrid formulation and native apigenin administration; therefore, Equation (1) can be written as Equation (2).

## 4. Conclusions

Triple antiplatelet therapy is considered as an efficient tool in the secondary prevention of atherothrombosis in patients with an acute myocardial infraction. However, the combinatorial use of three different antiplatelet drugs can lead to adverse effects, including the high risk of bleeding. Our group determined to identify a bioactive compound able to inhibit the three platelet activation pathways and in in silico studies, identified that apigenin could simultaneously inhibit PAR-1, P2Y12, and COX-1 platelets. However, this method demonstrates low bioavailability, and consequently, reduced antiplatelet activity. To enhance apigenin’s antiplatelet activity profile, a novel hybrid of apigenin with DHA was designed by exploiting the beneficial properties of DHA against CVDs, termed 4′-DHA-apigenin. During these experiments, it was recorded that the new hybrid exhibited inhibitory activity against all three platelet aggregation pathways. Importantly a 2-fold enhancement was determined by 4′-DHA-apigenin for TRAP-6 and AA, with respect to native apigenin. Additionally, a 12-fold enhancement was recorded by the hybrid for TRAP-6, with respect to DHA. This pinpoints that the beneficial properties of two isolated components can successfully result in a new hybrid exhibiting an enhanced profile with respect to the parent components.

To enhance the UHPLC-MS/MS recorded low plasma stability of the molecular hybrid, as well as its associated efficacy, an olive oil-based dosage form was developed, using olive oil as a vehicle. Interestingly, the new formulation demonstrated enhanced antiplatelet activity with respect to the parent hybrid. The increased triple antiplatelet profile of the developed formulation could be attributed to the increased stability and the beneficial properties of olive oil. The in vivo pharmacokinetic profile of formulations of both apigenin (dissolved in extra virgin olive oil) and 4′-DHA-apigenin (as an olive oil-based formulation) after oral administration in the C57BL/6J mice model were evaluated. The oral absorption of the 4′-DHA-apigenin olive oil formulate was more rapid and more extensive than that of the apigenin formulation, with a relative bioavailability of 262%. This result further capitalizes on the potential for developing olive oil-based dosage forms utilizing polyunsaturated fatty acids (omega-3 PUFA) and plant secondary metabolites to combat ASCVD and complement the modern triple antiplatelet regimen.

## Figures and Tables

**Table 1:** IC50 values of the inhibitory effect of 4′-DHA-apigenin hybrid, apigenin/DHA (equimolar solution), apigenin and DHA on AA, ADP and TRAP-6-induced platelet aggregation.

Compounds	AA	ADP	TRAP 6
	IC50 (μM)	IC50 (μM)	IC50 (μM)
4′-DHA-apigenin hybrid	353 ± 36 *	362 ± 20 ^§^	52 ± 12 ^#^
i. Apigenin/DHA equimolar solution	210 ± 25	580 ± 39	610 ± 40
ii Apigenin	850 ± 45	607 ± 35	56 ± 8
iii. DHA	130 ± 18	920 ± 53	680 ± 42

The values are the mean ± SD from three different platelet preparations. *^, §, #^ *p* < 0.005, compared with the inhibitory effect of: (i) apigenin/DHA equimolar solution, on platelet aggregation induced by AA, ADP and TRAP-6, (ii) apigenin, on platelet aggregation induced by AA and ADP, and (iii) DHA, on platelet aggregation induced by ADP and TRAP-6, respectively.

**Table 2:** IC50 values of the inhibitory effect of 4′-DHA-apigenin hybrid/olive oil formulation, 4′-DHA-apigenin hybrid and olive oil, on AA, ADP and TRAP-6-induced platelet aggregation.

Compounds	AA	ADP	TRAP-6
	IC50 (μM)	IC50 (μM)	IC50 (μM)
Formulate (olive oil-4′-DHA-apigenin hybrid)	306 ± 18 *	644 ± 35 ^§^	16 ± 3 ^#^
(i) 4′-DHA-apigenin hybrid	353 ± 36	362 ± 20	52 ± 12
(ii) Olive oil	>1000	820 ± 45	>1000

The values are the mean ± SD from three different platelet preparations. *^, §, #^ *p* < 0.005, compared with (i) 4′-DHA-apigenin hybrid and (ii) Olive oil inhibitory effect on platelet aggregation induced by AA, ADP and TRAP-6.

**Table 3:** Serum PK parameters of apigenin and 4′-DHA-apigenin after oral administration.

PK Parameter	Apigenin **	4′-DHA-Apigenin #
	Estimate (%CV)	Estimate (%CV)
T_max_ (h)	2	2
C_max_ (μg/L)	10.8 (1.09)	17.8 (4.56)
AUC_0−t_ (h × μg/L)	34.97 (6.73)	91.7 (5.11)
AUC_inf_ (h × μg/L)	58.75	471
AUC % extrapolated	40	80
t_1/2_ (h)	3.44	17.4
Vz/F (L) *	105.5	66.5
Cl/F (L/h) *	21.3	2.65
Relative bioavailability %		262

* Vz (volume of distribution) and Cl (clearance) estimates are normalized with respect to the fraction of the dose absorbed (F); ** administered orally as dissolved in olive oil; # administered orally as an olive oil-based formulation.

## Data Availability

Data is contained within the article or Supplementary Material.

## Supplementary Materials

The following supporting information can be downloaded at: https://www.mdpi.com/article/10.3390/molecules28052311/s1, Table S1. Hydrogen bonds, Hydrophobic interactions, pi interactions, Potential Energy (OPLS3) and XP Gscore for Apigenin with each receptor. Table S2. Hydrogen bonds, Hydrophobic interactions, pi interactions, Potential Energy(OPLS3) and XP Gscore for 4′-DHA-apigenin with each receptor. Figure S1. Structure of 4′-DHA-apigenin. Figure S2. ^1^H-NMR of 4′-DHA-apigenin. Figure S3. 13C-NMR of 4′-DHA-apigenin. Figure S4. MS spectra of 4′-DHA-apigenin.
