# Supplementary material for: Development of a Novel Apigenin Dosage form as a Substitute for the Modern Triple Antithrombotic Regimen
Source: Molecules. 2023 Mar 2;28(5):2311. doi: 10.3390/molecules28052311 (PMC10005222; doi:10.3390/molecules28052311)
Supplement: Supplementary file 1 [file molecules-28-02311-s001.zip › molecules-2225081-supplementary.pdf]

Development of a Novel Apigenin Dosage form as a Substitute for the Modern Triple Antithrombotic Regimen

<u>Figures and Tables:</u>

Table S1: Hydrogen bonds, Hydrophobic interactions, pi interactions, Potential Energy (OPLS3) and XP Gscore for Apigenin with each receptor.

RECEPTORS	HYDROGEN BONDS	HYDROPHOBIC INTERACTIONS	Pi INTERACTIONS	OPLS3	XP GScore
PAR-1	ASP 256	TYR 350<br>ALA 349<br>ALA 352<br>TYR 353<br>LEU 332<br>LEU 333<br>LEU 340	TYR 353	-586,53	-8.57
P2Y12	CYS 175<br>GLU 281<br>ARG 93<br>TYR 105	PHE 277<br>CYS 175<br>TYR 105<br>VAL 102	HIS 187<br>ARG 256	-586.52	-4.75
COX-1	TYR 385<br>SER 530	VAL 116<br>LEU 531<br>VAL 349<br>ILE 523<br>LEU 352<br>TYR 385<br>TRP 387	ARG 120	-586.52	-5.36

Table S2: Hydrogen bonds, Hydrophobic interactions, pi interactions, Potential Energy(OPLS3) and XP Gscore for 4´-DHA-apigenin with each receptor.

RECEPTORS	HYDROGEN BONDS	HYDROPHOBIC INTERACTIONS	Pi INTERACTIONS	OPLS3	XP GScore
PAR-1	TYR 350<br>LEU258<br>TYR 337	TYR 353<br>LEU 237<br>LEU 333<br>LEU 263<br>LEU 262<br>TYR 267<br>TYR 270<br>PHE 271<br>PHE 274<br>PRO 236		-833.26	-13.461
P2Y12	ARG 93<br>CYS 175<br>GLN 263<br>TYR 259	LEU 261<br>ILE 257<br>TYR 192<br>TYR 109<br>CYS 194<br>PHE 252<br>VAL 190<br>TYR 105<br>CYS 175<br>PHE 277	LYS 280<br>ARG 256<br>HIS 187	-828.489	-1.93
COX-1	ILE 517<br>SER 516	ILE 517<br>PHE 518<br>ILE 523<br>TYR 355<br>VAL 116<br>ALA 527<br>LEU 531<br>VAL 349<br>LEU 359<br>TYR 385<br>TRP 387<br>PHE 381		-828.489	-9.250

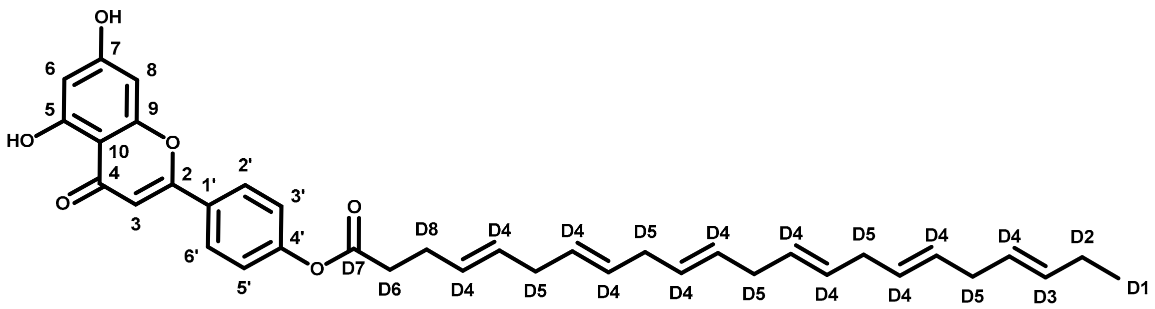

Figure S1. Structure of 4´-DHA-apigenin

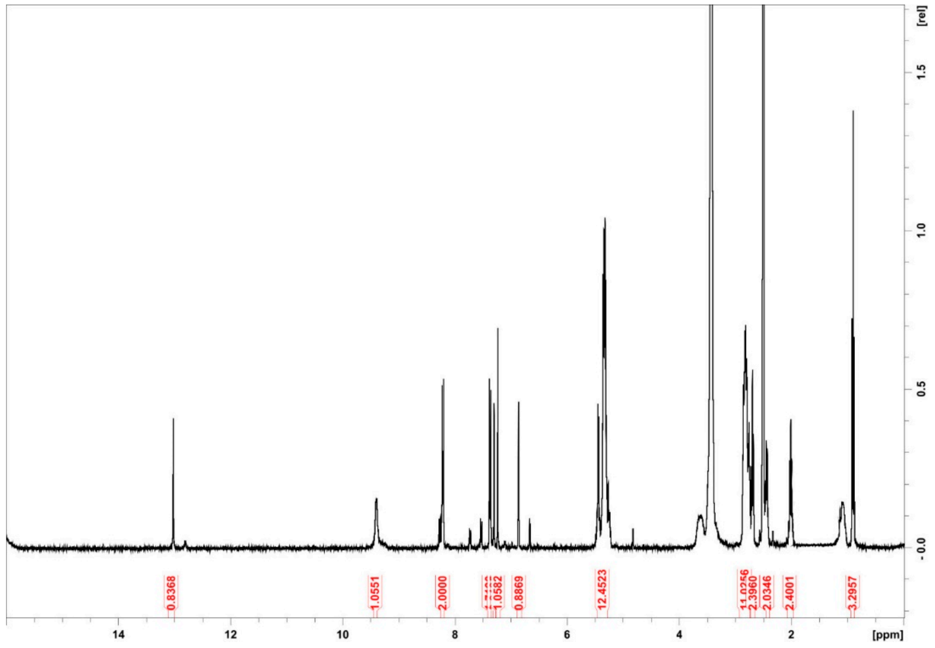

Figure S2. ^{1}H-NMR of 4´-DHA-apigenin.

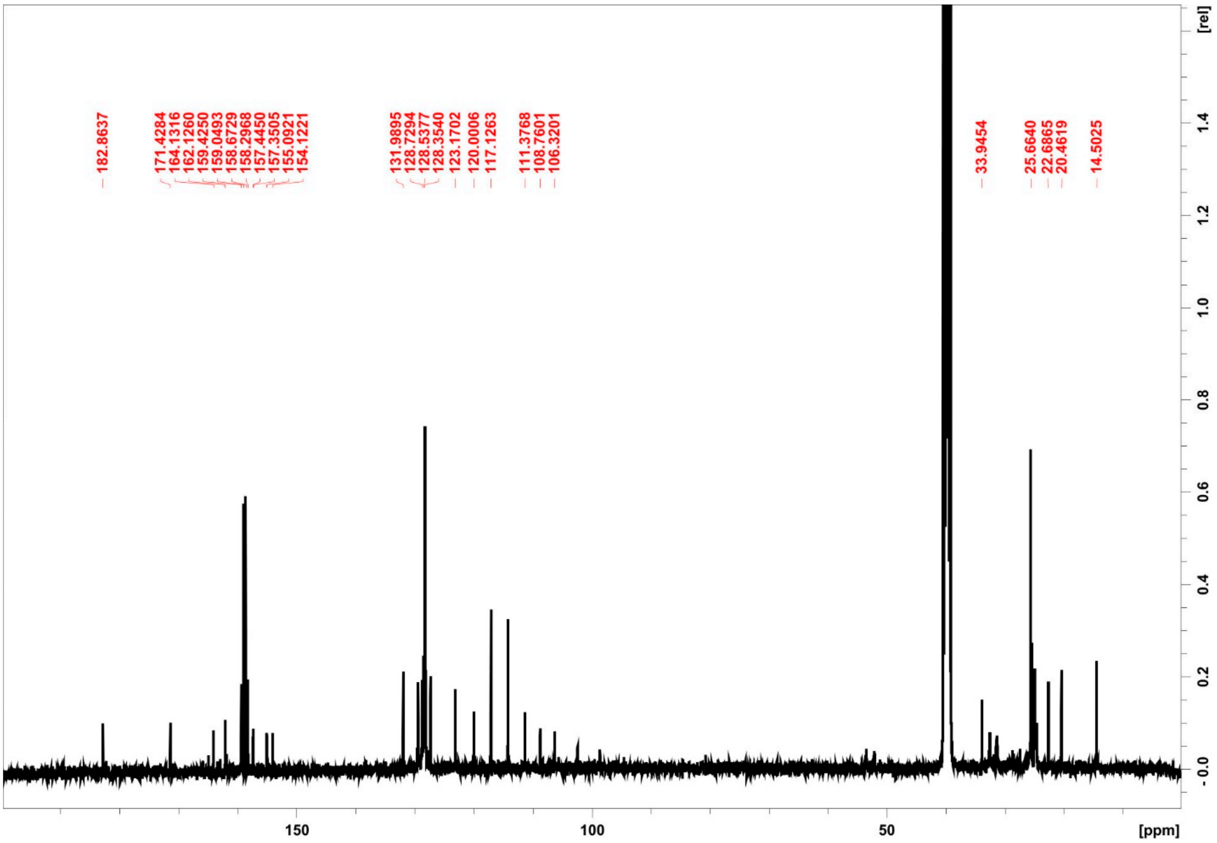

Figure S3. ^{13}C-NMR of 4´-DHA-apigenin.

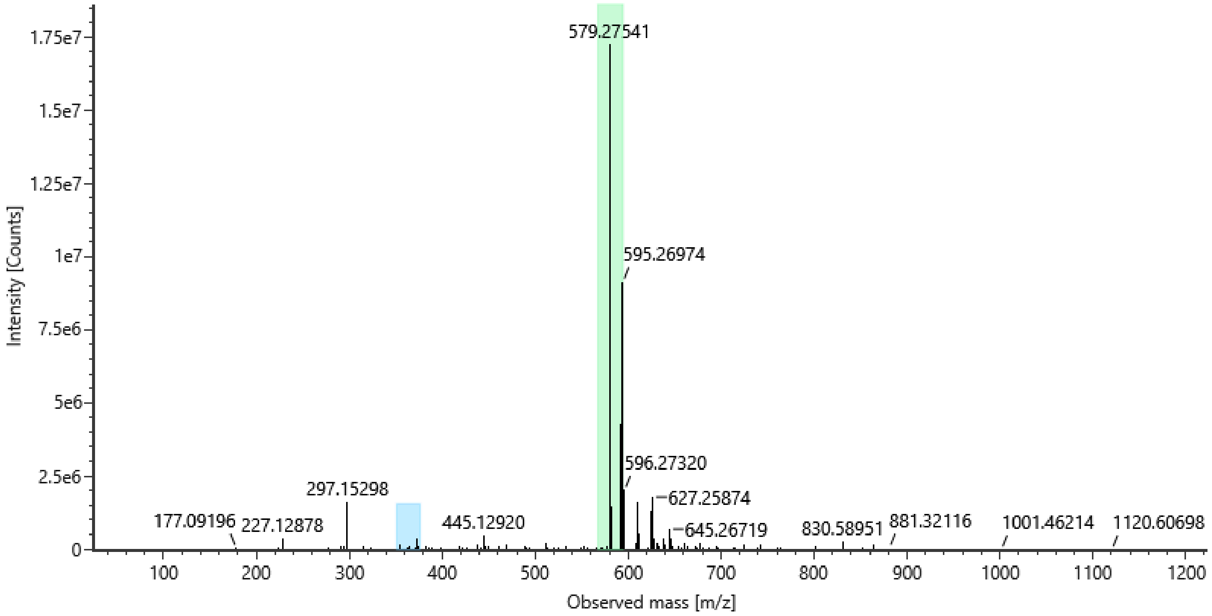

Figure S4. MS spectra of 4´-DHA-apigenin.
